# Selection of Shared and Neoantigen-Reactive T Cells for Adoptive Cell Therapy Based on CD137 Separation

**DOI:** 10.3389/fimmu.2017.01211

**Published:** 2017-10-10

**Authors:** Sivan Seliktar-Ofir, Efrat Merhavi-Shoham, Orit Itzhaki, Sharon Yunger, Gal Markel, Jacob Schachter, Michal J. Besser

**Affiliations:** ^1^Ella Lemelbaum Institute for Immuno-Oncology, Sheba Medical Center, Tel Hashomer, Israel; ^2^Department of Clinical Immunology and Microbiology, Sackler School of Medicine, Tel Aviv University, Tel Aviv, Israel

**Keywords:** adoptive cell therapy, melanoma, tumor infiltrating lymphocytes (TIL), CD137, neoantigen, tumor antigen

## Abstract

Adoptive cell therapy (ACT) of autologous tumor infiltrating lymphocytes (TIL) is an effective immunotherapy for patients with solid tumors, yielding objective response rates of around 40% in refractory patients with metastatic melanoma. Most clinical centers utilize bulk, randomly isolated TIL from the tumor tissue for *ex vivo* expansion and infusion. Only a minor fraction of the administered T cells recognizes tumor antigens, such as shared and mutation-derived neoantigens, and consequently eliminates the tumor. Thus, there are many ongoing effects to identify and select tumor-specific TIL for therapy; however, those approaches are very costly and require months, which is unreasonable for most metastatic patients. CD137 (4-1BB) has been identified as a co-stimulatory marker, which is induced upon the specific interaction of T cells with their target cell. Therefore, CD137 can be a useful biomarker and an important tool for the selection of tumor-reactive T cells. Here, we developed and validated a simple and time efficient method for the selection of CD137-expressing T cells for therapy based on magnetic bead separation. CD137 selection was performed with clinical grade compliant reagents, and TIL were expanded in a large-scale manner to meet cell numbers required for the patient setting in a GMP facility. For the first time, the methodology was designed to comply with both clinical needs and limitations, and its feasibility was assessed. CD137-selected TIL demonstrated significantly increased antitumor reactivity and were enriched for T cells recognizing neoantigens as well as shared tumor antigens. CD137-based selection enabled the enrichment of tumor-reactive T cells without the necessity of knowing the epitope specificity or the antigen type. The direct implementation of the CD137 separation method to the cell production of TIL may provide a simple way to improve the clinical efficiency of TIL ACT.

## Introduction

Adoptive cell therapy (ACT) of tumor infiltrating lymphocytes (TIL) is an effective therapy for patients with metastatic melanoma (1), leading to objective response rates of 40–50%, with some of those patients experiencing durable complete regression of their metastatic tumors (2–4). This form of ACT utilizes T cells that are isolated from fresh tumors, rapidly expanded and reactivated *ex vivo* and then transferred back into the patient to eliminate the cancer cells (2, 5–8).

T cell responses rely on T cell receptor (TCR)-mediated recognition of tumor antigen derived from shared tumor-associated antigens (TAA) or neoantigens presented by self-MHC molecules (9–15). Neoantigenic peptides arise from somatic mutations occurring during neoplastic transformation and are mostly tumor, and even patient specific. The presence of tumor-specific MHC-neoantigen complexes on the surface of malignant cells represents a unique and specific target for T cells (16, 17). Shared/TAA, such as NY-ESO-1, MART-1, and gp100, are typically over-expressed in malignant cells, but also exist in normal cells (10–12).

T cells that target tumor neoantigens have been suggested to be the main mediators of effective cancer immunotherapies, not only in the context of adoptive T cell therapy, but also for successful treatment with checkpoint modulators against CTLA-4 and PD-1 (18, 19). Neoantigen-reactive TIL have been identified in the infusion products of metastatic melanoma patients who achieved durable cancer regression following ACT. As a result, multiple research efforts are currently being invested in the identification and selection of tumor mutation-specific TIL for therapy (20–22); however, these approaches are still very complex. Whole-exome sequencing (WES) of tumor DNA in combination with RNAseq and *in silico* HLA-binding prediction has been applied to identify non-synonymous cancer mutations recognized by T cells. This analysis can result in dozens or even hundreds of potential candidate peptides in highly mutated tumor types, such as melanoma (20, 22, 23). Candidate peptides, tetramers or tandems of minigenes (TMG) of those peptides are then expressed on MHC matched antigen-presenting cells (APC) and co-incubated with TIL cultures (22, 24). Neoantigen-reactive T cell cultures can be identified, as they specifically secrete interferon (IFN) γ or upregulate co-stimulatory molecules, such as CD134OX-40 or CD137 (4-1BB) upon peptide recognition (17, 25).

We have recently developed an alternative analytical tool that combines WES with HLA peptidome mass spectrometry, to identify neoantigenic peptides that are actually processed and presented by the tumor HLA molecules (26). Although the latter method is already more cost and time effective, all approaches still require sophisticated equipment and a period of several months. For most metastatic patients, this timeframe is unreasonable. Therefore, a quick and easy method for the identification of antitumor-reactive TIL is required, to make this approach clinically applicable.

Following antigen recognition, T cells undergo a wide range of phenotypic and functional changes including cytokines secretion and upregulation of multiple activation markers such as CD25, CD38, and CD69. The specific upregulation of co-stimulatory molecules, such as CD137 or CD134 or co-inhibitory molecules, such as CD279 (PD-1), provides an opportunity of using those molecules as biomarkers to detect and select tumor-reactive T cells for therapy (18, 27, 28).

CD137, a member of the TNF receptor superfamily, is an activation induced T cell co-stimulatory molecule. Signaling with CD137 upregulates survival genes, enhances cell division, induces cytokine production and prevents activation induced cell death of T cells. Others and our institute have shown that T cells co-incubated with APC loaded with neoantigenic or shared tumor peptides upregulate CD137 expression (21, 29, 30). This upregulation is highly specific and occurs only in T cells that recognize the antigenic tumor peptide. Ye et al. evaluated the immunobiology of CD137 in human ovarian cancer and showed that CD137^+^ T cells when cocultured with autologous tumor cells, demonstrated increased reactivity against shared antigens (31). Importantly, this fraction of cells demonstrated enhanced *in vitro* and *in vivo* antitumor reactivity. Recently, Parkhurst et al. isolated CD137^+^ TIL by FACS sorting after stimulation with dendritic cells transfected with mutated TMG RNA and could show that expanded CD137^+^ cells are enriched for neoantigen-specific T cells (32). TCRs, isolated from those cells, were then introduced into autologous peripheral blood lymphocytes (PBL) to induce tumor rejection *in vitro*. Therefore, CD137 serves as a potential biomarker to isolate shared, as well as neoantigen-reactive TIL.

Here, we describe systematically a novel and time efficient method for the identification and selection of CD137-expressing T cells for therapy based on magnetic bead separation, using clinical grade compliant reagents. The method was adjusted to meet clinical limitations and needs. As expected, CD137-selected TIL showed significantly increased antitumor reactivity. This approach can be directly implemented to the clinical cell production of TIL and may provide a simple way to improve the efficiency of TIL ACT.

## Materials and Methods

### Generation of TIL and Autologous Melanoma Lines

TIL and autologous melanoma lines were isolated from tumor biopsies of metastatic melanoma patients, enrolled to a phase II TIL ACT trial at the Sheba Medical Center (NCT00287131). Patients signed an informed consent approved by the Israeli Ministry of Health (Helsinki approval no. 3518/2004), which allowed enrollment to the clinical trial and the use of excess cell material for research purpose. The generation of TIL was conducted precisely as for the clinical setting and has been described in detail before (5). In short, fragmentation, enzymatic digestion, fine needle aspiration (FNA), and tissue remnant culture (TRC) techniques were used to isolate TIL and melanoma cells from surgically resected metastatic lesions. TIL were cultured in complete medium (CM) containing 10% human serum (Cat. no. 100-512, Gemini Bio-Products), 25 mmol/l HEPES (Cat. no. 03-025-1B, Biological Industries), 100 U/ml penicillin, 100 µg/ml streptomycin (penicillin–streptomycin mixture, Cat. no. BE-17-602E, Lonza), 50 µg/ml gentamicin (Cat. no. PZN-3928180, TEVA Pharmaceutical), and 5 × 10e (−5) M 2-mercaptoethanol (Cat. no. 31350-010, Thermo Fisher Scientific) in RPMI 1640 (Cat. no. 12-702F, Lonza). Melanoma cells were cultures in MEL medium contained 10% human serum, 25 mmol/l HEPES, 100 U/ml penicillin, 100 µg/ml streptomycin and 2 mmol l-glutamine (Cat. no. BE-17-605E, Lonza), and 1 mM sodium pyruvate (Cat. no. BE-13-115E, Lonza) in RPMI 1640. TIL cultures were *ex vivo* expanded in three major steps: (1) pre-rapid expansion procedure (Pre-REP); TIL were cultured in 24-well plates, and CM with 3,000 IU/ml IL-2 (Cat. no. CH1001, Proleukin, Novartis Pharmaceuticals) was added every 2–3 days to keep the cell concentration at 0.5–2 × 10e6/ml. TIL cultures typically reached a total number of about 200 × 10e6 within 3–4 weeks, (2) CD137 selection; established TIL were cocultured with autologous melanoma cells followed by a magnetic bead separation of CD137-expressing cells; detailed below, (3) REP; unselected or CD137-selected TIL were expanded in a large-scale expansion procedure utilizing 30ng/ml anti-CD3 GMP antibody (Cat. no. 176-076-116; Miltenyi), 3,000 IU IL-2, and 50 Gy irradiated feeder cells of healthy donors (feeder cells to TIL = 100:1) in GRex flasks as detailed in Besser et al. (2). Feeder cells consist of a mixture, approximately equal proportions, of peripheral blood mononuclear cells (PBMC) isolated by Ficoll gradient from the apheresis product of three healthy donors. The intention is to compensate for a possible poor source of feeder cells. Within 14 days, cultures expanded by about 1,000-fold. Day 14 is the potential day of infusion.

### Generation of Autologous EBV Transformed B Cell Lines

PBL were separated by centrifugation on a Ficoll/Hypaque cushion. 10e7 mononuclear cells were centrifuged at 400 *g* for 10 min, and supernatant was carefully aspirated; the cells were resuspended in 1 ml B cell medium (10% human serum, 100 U/ml penicillin, 100 µg/ml streptomycin, and 2 mmol l-glutamine in RPMI 1640), and 1 ml of Epstein-Barr virus (EBV) particles (kindly provided by Prof. E. Gazit—Sheba Medical Center, Israel) was added in a 15 ml conical tube. After careful mixing, the cells were incubated for 2 h at 37°C. Another 3 ml of B cell medium containing 1 µg/ml cyclosporine (Cat. no. tlrl-cyca, InvivoGen) were then added to the tube. The cells were transferred to a 25 cm^2^ tissue culture flask and placed horizontally in an incubator at 37°C in a 5% CO_2_ atmosphere for 3 weeks. Fresh B cell medium with cyclosporine was added whenever the medium turned yellow.

### Coculture and CD137 Separation

After completion of the Pre-REP step, TIL were cocultured with autologous melanoma cells at an effector to target (E:T) ratio of 8:1 (or as indicated). For this purpose 0.5 × 10e6 melanoma cells were resuspended in 2 ml MEL medium and transferred to one well of a 24-well plate. Typically, 48 wells (24 × 10e6 melanoma cells) were set up per experiment. Plates were incubated overnight at 37°C to allow adhesion of the tumor cells. The following day plates were rinsed with RPMI to remove non-adhered tumor cells. 4 × 10e6 TIL (in 2 ml MEL medium) were added per well and co-incubated with the tumor cells at 37°C. After 6–8 h (or as indicated), TIL were collected, centrifuged at 300 *g* for 10 min, and resuspended in CliniMACS PBS/EDTA buffer (Cat. no. 700-25, Miltenyi Biotech). To determine the number of CD137^+^ cells, a small sample of the cells was removed to perform CD137 flow cytometry (detailed below). In the meantime, cells were centrifuged, and the pellet was resuspended with biotinylated anti-CD137 antibody (Cat. no. 701-30, CliniMACS CD137-Biotin reagent, Miltenyi Biotech, CE approved) (37.5 µl anti-CD137 antibody in 1 ml CliniMACS Buffer per 5 × 10e6 total cells), independent of the frequency of CD137^+^ cells. The cell suspension was incubated for 30 min in the dark at 2–8°C and washed by adding 10 ml of CliniMACS PBS/EDTA buffer and centrifugation (300 *g* for 10 min). The cell pellet was resuspend with CliniMACS Anti-Biotin Reagent (Cat. no. 173-01, Miltenyi Biotech, CE approved) (37.5 µl anti-biotin MicroBeads in 1 ml CliniMACS Buffer per 5 × 10e6 total cells), incubated for 30 min in the dark at 2–8°C and washed by adding 10 ml of CliniMACS PBS/EDTA buffer followed by centrifugation (300 *g* for 10 min).

During the second incubation step, MACS ART MS Columns (Cat. no. 200-070-500; Miltenyi Biotech, CE approved) were prepared by connecting the columns to the MACS ART Separation Unit (Cat. no. 200-070-501, Miltenyi Biotech, see the manufacture’s instruction; CE approved) and rinsing each column with 500 µl of MACS buffer. Results of the CD137 flow cytometry were obtained.

According to the manufacture’s instruction, the capacity of one MS ART column is 10e7-labeled cells and up to 20e8 total cells. The cell pellet was resuspended in 500 µl MACS buffer for up to 10e8 total cells and applied onto the prepared column.

Unlabeled, CD137^−^ cells passing through the column were collected after rinsing the column ones with 500 µl CliniMACS Buffer, and CD137^+^ cells were collected after removal of the column from the magnetic separator and rinsing the column twice with 500 µl CliniMACS Buffer (see the manufacture’s instruction).

### Coculture of TIL with Autologous EBV Transformed B Cell Lines (B-LCL)

B-LCL were resuspended in B cell medium at concentration of 2 × 10e6 per ml. 2 × 10e5 B-LCL were seeded in a well of a 96-well plates, and 5 µl of 25-mer peptide (stock: 0.2 mg/ml) was added. After an overnight incubation at 37°C, B-LCL were washed twice by centrifugation at 300 *g* for 6 min and resuspended in 100 µl MEL medium. A coculture was performed by adding 0.5 × 10e5 T cells suspended in 100 µl MEL medium to each well, followed by an overnight incubation at 37°C.

### Flow Cytometry

The following antibodies were used for flow cytometry: APC-labeled antihuman CD137 (Cat. no. 130-094-821, clone 4B4-1, Miltenyi Biotech), FITC-labeled antihuman CD4 FITC (Cat. no. PMG555346 BD Bioscience), PE-Cy7-labeled antihuman CD8 (Cat. no. 344712, BioLegend), APC-labeled antihuman CD45 (Cat. no. 340910 BD Bioscience), PE-labeled anti-MCSP (Cat. no. 130-091-225 Miltenyi Biotec), and PE-labeled antihuman CD3 (Cat. no. 555340 BD Bioscience).

TIL were washed and resuspended in FACS buffer consisting of 0.5% BSA in PBS. Cells were incubated for 30 min with the antibodies on ice, washed in FACS buffer, and read as the relative log fluorescence of live cells using MACSQuant flow cytometer (Miltenyi Biotech). Samples were analyzed using FlowJo software (BD Bioscience). Cells were gated on viable lymphocytes, according to FSC and SSC, as well as singlets. If indicated, the cells were further gated on CD3 T cells (Figure S1 in Supplementary Material).

### IFNγ Release Assay

TIL were cocultured overnight with autologous melanoma cells in 96-well plates at an E:T ratio of 1:1 (5 × 10e5 each in a total of 200 µl MEL medium) or as indicated. Cells were centrifuged, supernatant was collected, and secreted IFNγ levels were determined by sandwich enzyme-linked immunosorbent assay (ELISA) according to the manufacturer’s instructions (Cat. no. 430104, BioLegend). Measurements were performed in triplicates.

### Cell-Mediated Cytotoxicity Assay

TIL were cocultured with autologous melanoma cells overnight at 37°C, at an E:T ratio of 1:1 (1×10e5 each in a total of 200 µl MEL medium). Cells were centrifuged, supernatant was collected, and the levels of lactate dehydrogenase (LDH), a stable cytosolic enzyme that is released upon cell lysis, were determined by CytoTox 96^®^ Assay according to the manufacturer’s instructions (Cat. no. G1780, Promega). Measurements were performed in triplicates.

### Statistical Analysis

Significance of variation between groups was evaluated using a non-parametric two-tailed Student’s *t*-test. Test for differences between proportions was performed using two-sided Fisher’s exact test with *p* ≤ 0.05 considered significant and *p* ≤ 0.01 highly significant. To strengthen the trend of dependence between two kinds of variables, we used the Spearman rank correlation coefficient.

## Results

### Establishment of TIL and Autologous Melanoma Lines

Twelve metastatic melanoma patients, age 49 ± 14 years, underwent resection of a metastatic lesion. TIL isolation and generation was performed precisely as for the clinical setting (5–7).

Baseline characteristics, origin of metastasis and the size of metastasis are summarized in Table 1. Fragmentation (typically 12 fragments per patient), enzymatic digestion, fine needle aspiration (FNA) and tissue remnant culture (TRC) techniques were applied to generate TIL and melanoma cultures from surgically resected metastatic lesions (2, 5–7). In one patient, TIL and melanoma cultures could not be established (patient #12) and in three more patients autologous melanoma cells failed to grow (patients #9, #10, and #11, Table 1). Those four patients were excluded from further analysis. For the other eight patients (patients #1–#8), an average of 304 × 10e6 ± 87 × 10e6 TIL was established in an average of 14 days (range 7–21 days).

**Table 1:** Baseline characteristics and CD137 expression of TIL patients.

Pt.	Age/gender	Origin of Met.	Size (cm^3^)	Days and method to reach at least 200 × 10e6 TIL in total	% CD8^+^	Autol. mel. availed	CC feasible^a^	CD137 expression before CC (%)	CD137 expression after CC (%)
1	57/M	SC	2.3	Day 14 (total 301 × 10e6) Digest 130 × 10e6 TRC 63 × 10e6 Frag. 108 × 10e6 FNA not performed	99	Yes	Yes	1.4	72.3
2	25/M	LN	1.4	Day 7 (total 325 × 10e6) Digest 300 × 10e6 TRC 25 × 10e6 Frag. None FNA none	55	Yes	Yes	4.2	17.1
3	41/M	SC	7.8	Day 11 (total: 406 × 10e6) Digest 320 × 10e6 TRC 70 × 10e6 Frag. 10 × 10e6 FNA 6 × 10e6	92	Yes	Yes	3.0	43.2
4	61/F	SC	6.1	Day 21 (total 200 × 10e6) Digest 120 × 10e6 TRC None Frag. 80 × 10e6 FNA none	40	Yes	Yes	8.3	14.1
5	41/M	SC	6.4	Day 17 (total 240 × 10e6) Digest 50 × 10e6 TRC: 50 × 10e6 Frag. 140 × 10e6 FNA none	87	Yes	Yes	2.3	3.3
6	50/M	Visceral	5.2	Day 14 (total 432 × 10e6) Digest 25 × 10e6 TRC: 250 × 10e6 Frag. 25 × 10e6 FNA: 132 × 10e6	84	Yes	Yes	3.8	33.7
7	36/M	LN	20	Day 13 (total 231 × 10e6) Digest 14 × 10e6 TRC 159 × 10e6 Frag. 47 × 10e6 FNA 11 × 106	76	Yes	Yes	3.6	56.8
8	45/M	SC	34	Day 17 (total 248 × 10e6) Digest 85 × 10e6 TRC 21 × 10e6 Frag. 142 × 10e6 FNA none	54	Yes	Yes	0.3	0.5
9	74/M	LN	162	Day 13 (total 350 × 10e6) Digest 100 × 10e6 TRC 110 × 10e6 Frag. 100 × 10e6 FNA 40 × 106	75	No	No	nd	nd
10	64/M	visceral	8.0	Day 13 (total 246 × 10e6) Digest 158 × 10e6 TRC 26 × 10e6 Frag. 32 × 10e6 FNA 30 × 106	96	No	No	nd	nd
11	50/F	SC	16	Day 25 (total 202 × 10e6) Digest 126 × 10e6 TRC 32 × 10e6 Frag. 32 × 10e6 FNA none	98	No	No	nd	nd
12	61/F	SC	3.8	Day 23 (not reached) Digest 15 × 10e6 TRC 2 × 10e6 Frag. 3 × 10e6 FNA none	nd	No	No	nd	nd

*Met., metastasis; Autol. mel., autologous melanoma line; CC, coculture; M, male; F, female; LN, lymph node; SC, subcutaneous; Digest, enzymatic digestion; TRC, tissue remnant culture; Frag., fragments (cell number indicates the total sum of TIL in 6–12 initiated fragment cultures); FNA, fine needle aspiration; nd, not determined*.

*% CD8^+^ within the CD3^+^ T cell population (all others are CD4^+^ cells)*.

*^a^A coculture was considered feasible when a minimum total number of 200 × 10e6 TIL was available and autologous melanoma lines were successfully established*.

Retrospective analyses of TIL and melanoma cultures in 203 patients enrolled between the years 2005 and 2016 to our TIL ACT trial, showed that in 20 (10%) of the patients TIL numbers of over 200 × 10e6 TIL could not be reached and in 97 (48%) of the patients autologous melanoma lines could not be established within 1–2 months (or both). Since the co-incubation of both cell types is required prior to CD137 separation (see below), the method seems feasible for about half of enrolled patients.

### CD137 Expression and Correlation to *In Vitro* Reactivity

Baseline CD137 expression in established TIL cultures before and following co-incubation with autologous melanoma lines was determined in patients #1–#8. For each patient, one of multiple TIL cultures or a pool of different TIL cultures were analyzed. As expected, baseline CD137 expression was low in TIL cultures before coculture (3.4 ± 2.4%) or TIL cultures co-incubated with HLA-mismatched melanoma lines (data not shown) and significantly increased after coculture with autologous melanoma lines (30.1 ± 25.9%; *p* = 0.011) (Table 1), supporting the understanding that CD137 serves as a marker for tumor recognition.

**Figure 1:**
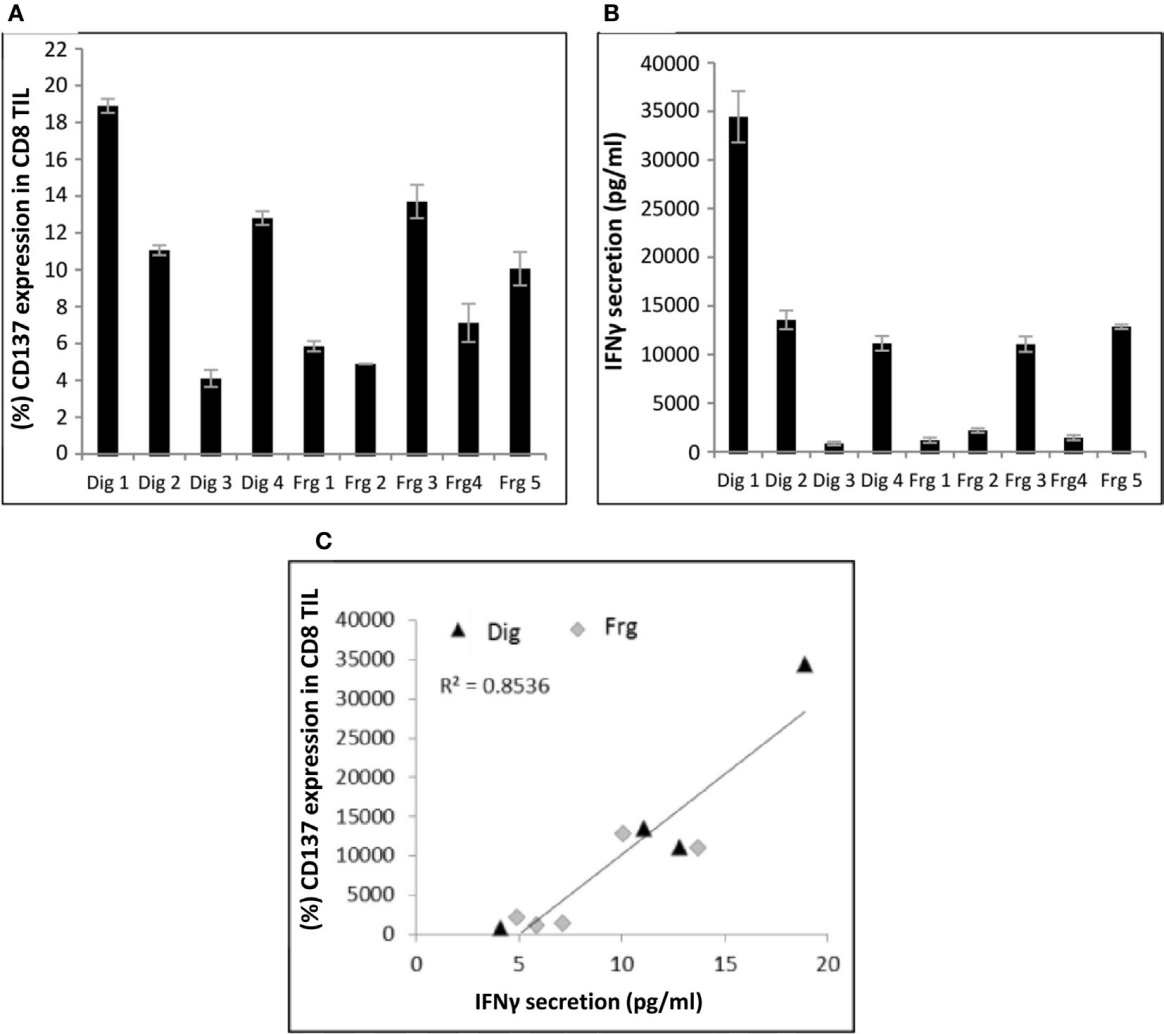
Correlation between CD137 expression and interferon (IFN) γ secretion. Multiple TIL cultures derived from the same melanoma biopsy of patient #4 were cocultured overnight with autologous melanoma cells at an E:T ratio of 1:1 (10e5 cells each). CD137 expression **(A)** and IFNγ secretion **(B)** were measured. Experiments were performed in triplicates. Correlation between the two is shown in panel **(C)**. Dig, enzymatic digest; Frg, fragments.

Nine independently grown TIL cultures [four cultures derived from enzymatic digestion (Dig 1–4) and five cultures (Frg 1–5) derived from different fragments] were established from the same biopsy of patient #4 and analyzed for CD137 expression following co-incubation with autologous tumor cells. As shown in Figure 1A, even among different TIL cultures derived from the same patient, there is a large variation of CD137 expression. CD137 expression was significantly correlated with *in vitro* reactivity measured as IFNγ secretion following co-incubation with target cells (Figures 1B,C; *R*^2^ = 0.85, *p* ≤ 0.01).

### Adaptation of the Coculture Assay to the Clinical Setting

Coculture assays for experimental purposes are often conducted in 96-well plates at an effector to target ratio of 1:1 by mixing 1 × 10e5 TIL with 1 × 10e5 target cells (25).

Four major adaptations had to be to done to make this approach feasible for the clinical setting: (1) Decreasing the number of autologous tumor cells, as they are often the limiting factor in the TIL to tumor cell ratio; (2) Up scaling of the coculture assay; (3) Defining the time point of maximal CD137 expression; and (4) Separation of tumor cells from TIL after coculture, as CD137-selected TIL are directly used for large-scale expansion and sequential infusion.

**Figure 2:**
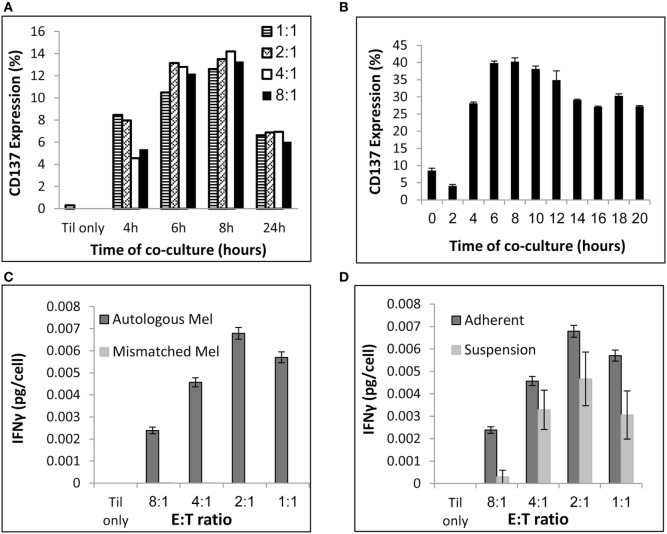
Coculture conditions. **(A)** Evaluation of CD137 expression after coculture at various E (TIL):T (melanoma) ratios and time points. **(B)** Evaluation of CD137 expression after coculture at E:T of 8:1 at various time points. **(C)** Interferon (IFN) γ secretion (pg/ml) per TIL cell following a 24 h coculture with autologous or HLA-mismatched melanoma cells at the indicated E:T ratios. **(D)** IFNγ secretion (pg/ml) per TIL cell following a 24 h coculture with autologous melanoma cells at the indicated E:T ratios. Melanoma cells were seeded 24 h before addition of TIL (“Adherent”) or simultaneously to TIL (“Suspension”). Experiments were performed in triplicates **(B,C,D)**.

To address those issues, pre-REPed TIL were cocultured overnight with autologous melanoma at different E:T ratios in 24-well plates by plating 0.5 × 10e6 tumor cells per well and addition of TIL at various E:T ratios in a final volume of 2 ml MEL medium. As shown before, for 90% of the patients TIL cultures can be established within 2–4 weeks, whereas melanoma cells grow often slowly. Therefore, we aimed to define an E:T ratio that requires low melanoma cell numbers. As shown in Figure 2A, E:T ratio of 8 TIL to 1 melanoma cell induced similar CD137 expression as for E:T of 1:1 (*p* = 0.3, normalized to 1:1 at the various time points). Figure 2A represents one of three experiments comparing different time points and E:T ratio, which all showed the same results. Consequently, cocultures can be conducted in two plates of 24-wells, by plating per well 0.5 × 10e6 melanoma cells and 4 × 10e6 TIL in a final volume of 2 ml media. This procedure requires altogether a total amount of 24 × 10e6 melanoma cells and 192 × 10e6 TIL cells in 96 ml medium.

To determine the time point of maximum CD137 expression, TIL were cocultured with target cells and CD137 expression was evaluated by flow cytometry at multiple time points. As shown in Figure 2B, the peak of CD137 expression was after 6–8 h.

As seen in Figure 2C, T cell reactivity measured by IFNγ secretion was detectable at all E:T ratios and specific, as HLA-mismatched melanoma did not induce cytokine secretion. The total concentration of IFNγ was obviously higher at 8:1 compared with 1:1, as those cultures contained eight times more TIL. To consider this difference, Figures 2C,D demonstrate IFNγ levels per T cell [measured as IFNγ secretion (pg/ml)/number of TIL]. Although the IFNγ levels per T cell was lower at E:T of 8:1 compared with 1:1 (*p* ≤ 0.004), an E:T of 8:1 was clearly sufficient to measure specific IFNγ secretion.

To avoid the passage over of tumor cells after coculture, melanoma cells were seeded for 16–24 h before addition of TIL to allow their attachment to the 24-well plate. Plates were rinsed twice with RPMI, to remove non-adhered tumor cell before adding TIL. As shown in Figure 2D, TIL cocultured with adhered melanoma cells secreted similar or higher IFNγ levels per T cell than TILs that were cocultured with non-adherent melanoma cells, which were added simultaneously with TIL (*p* ≤ 0.05 at 8:1 and *p* = 0.06 at 1:1). Importantly, immunocytology with hematoxylin/eosin stain and antibodies against pan-cytokeratin and CD45 was performed and analyzed by a certified pathologist on four post-REP TIL (the potential infusion product) and confirmed no evidence of melanoma cells (data not shown). This was further supported by FACS analysis with antibodies against the melanoma-associated chondroitin sulfate proteoglycan (MCSP) antigen and the leukocyte marker CD45 (data not shown). Immunocytology is the standard methodology of most regulatory bodies for the detection of cellular impurities. Theoretically, the end product (also without coculture and CD137 selection) could still contain tumor cells, although this is very unlikely due to the magnetic bead separation and culture conditions that strongly promote T cell growth and T cell-mediated killing of tumor cells.

In conclusion, the final coculture protocol was defined as follows: (1) transfer of 0.5 × 10e6 melanoma cells per 1 ml MEL medium to each well of two 24-well plates (total of 24 × 10e6 melanoma cells in 48 wells); (2) overnight incubation at 37°C; (3) rinse twice to remove non-adhered tumor cells; (4) add 4 × 10e6 TIL per 1 ml to each well (8:1 E:T ratio; resulting in a total of 192 × 10e6 TIL in 48 wells); (5) co-incubation for 6–8 h at 37°C; and (6) collection of TIL.

As shown in Table 1, the average frequency of CD137-expressing TIL following coculture was 30.1 ± 25.9%. Performing coculture in two 24-well plates (192 × 10e6 TIL in total) would therefore result in an average of 61 × 10e6 CD137-expressing TIL being availed for the next expansion step. In the clinical setting, the large-scale expansion of TIL (rapid expansion process, REP) is typically initiated with 30 to 50 × 10e6 TIL. For TIL cultures with a low frequency of CD137-expressing cells, coculture may be performed with additional 24-well plates.

### Magnetic Bead Separation of CD137-Expressing Cells

To allow the implementation of the CD137 selection process to the clinical setting, the enrichment process must be performed with clinical grade compliant reagents.

Following coculture, CD137-expressing cells were positively enriched by magnetic bead separation, by incubating 5 × 10e6 TIL first for 30 min with 37.5 µl/ml CliniMACS CD137-Biotin antibody, then for 30 min with 37.5 µl/ml CliniMACS Anti-Biotin Reagent and next transferred to MACS ART MS Columns, connected to a MACS ART Separation Unit (detailed in the Section “Materials and Methods”). Consequently, 192 × 10e6 TIL were incubated with 1.44 ml of each antibody and transferred to two to eight MACS ART MS Columns, depending on the percent of CD137-expressing cells following coculture (the column capacity is 1 × 10e7-labeled cells).

**Figure 3:**
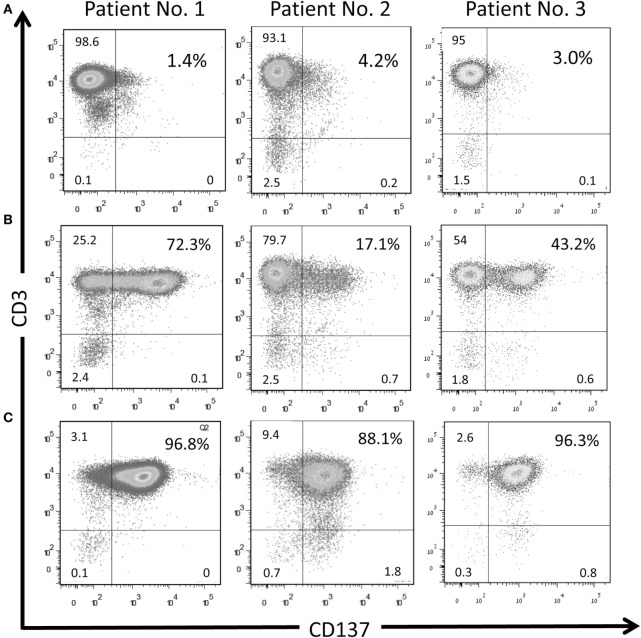
CD137 separation with magnetic beads enriches TIL population with activated TILs. CD137 expression on TIL cultures derived from three patients at baseline level **(A)**, after 6-h of co-incubation with autologous target cells (E:T 8:1) **(B)** and in the CD137^+^ fraction after magnetic bead separation **(C)**. Singlet cells were gated on lymphocytes according to FSC and SSC.

TIL cultures from three patients (patients #1–#3) were enriched for CD137-expressing cells. As shown in Figure 3, in those three patients the baseline level of CD137-expressing cells was 2.9 ± 1.4%, increased to 44.2 ± 27.6% after coculture and further increased to 93.3 ± 4.9% in the CD137^+^ fraction after magnetic bead separation. The frequencies of CD4^+^, CD8^+^, and PD1+ cells within CD3 gated T cells are shown in Table 2. Since all TIL cultures of patient #1 were exclusively CD8 T cells (≥98%), the CD4/CD8 analysis was also performed on TIL of patient #4. The coculture assay itself does not affect the CD4/CD8 content. As shown in Table 2, although not significant, the percentage of CD8^+^ cytotoxic T cells in the CD137 fraction was higher in all four patients compared with unseparated TIL; however, also the CD137^+^ fraction contained CD4^+^ T cells (16.6 ± 16.3%).

**Table 2:** CD8, CD4, and PD-1 frequency of CD137 separated cells.

	% CD8		% CD4		% PD-1		
	Before separation	CD137 fraction	Before separation	CD137 fraction	Before CC	After CC	CD137 fraction
Pt. 1	98.5	99.6	1.5	0.4	9.7	36.2	49.8
Pt. 2	54.6	71.1	45.4	28.9	36.1	45.1	42.3
Pt. 3	92.0	95.2	8.0	4.8	22.9	49.2	91.3
Pt. 4	39.9	67.9	60.1	32.1	nd	nd	nd
Av.	71.3 ± 28.5	83.5 ± 16.3	28.8 ± 28.5	16.6 ± 16.3	22.9 ± 13.2	43.5 ± 6.6	61.1 ± 26.4

*p*-Value	0.48		0.48		0.07		0.32

*CC, coculture with autologous melanoma cells; nd, not determined*.

*Cells were gated on viable, singlet CD3 T cells*.

**Figure 4:**
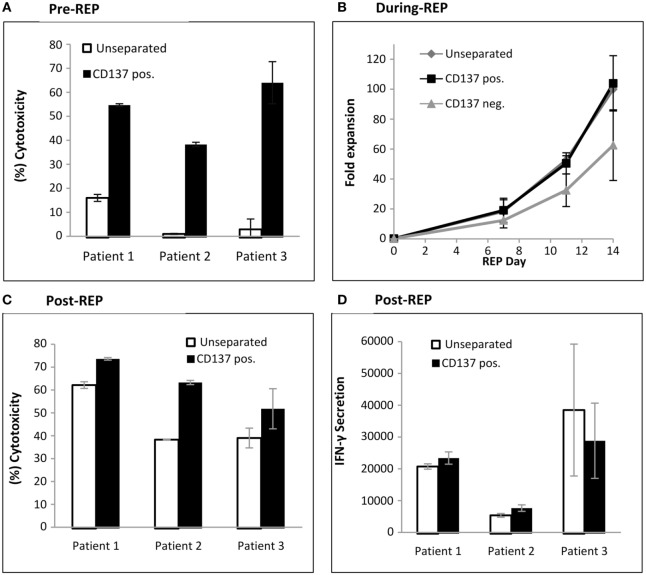
Antitumor reactivity and fold expansion of CD137 separated TIL compared with unseparated TIL. **(A)** Following magnetic bead separation, TIL of patients #1, #2, and #3 were cocultured with autologous melanoma cells and cytotoxicity was determined by lactate dehydrogenase (LDH) release. **(B)** Fold expansion of the CD137^+^ fraction, CD137^−^ fraction, and unseparated TIL during a 14-day rapid expansion procedure. Numbers were normalized to fold expansion on unseparated TIL on day 14. **(C,D)** *In vitro* reactivity of large-scale expanded TIL. On day 14 of REP, TIL were cocultured with autologous tumor cells, and cytotoxicity assay by LDH release **(C)** and interferon γ enzyme-linked immunosorbent assay (pg/ml) were performed **(D)**. All experiments were performed in triplicates.

The antitumor *in vitro* reactivity of TILs was determined post separation by a cytotoxicity assay. As shown in Figure 4A, the CD137^+^ fraction demonstrates significant increased antitumor reactivity compared with unseparated TIL (CD137^+^ fraction 52.3 ± 10.6%; unseparated 6.6 ± 6.6%; *p* ≤ 0.007).

### Large-scale Expansion of CD137 Selected TIL

To produce large numbers of TIL for infusion, TIL are expanded in a standard 14-day rapid expansion protocol resulting in the final drug product for infusion. Unseparated cells, CD137^+^ and CD137^−^ TIL from three patients were expanded. The fold expansions during REP, normalized to unseparated TIL on day 14 are shown in Figure 4B. The CD137^+^ fraction (103 ± 18%) reached on day 14 a similar fold expansion as unseparated TIL (100%; *p* = 0.78) or CD137^−^ TIL (63 ± 24%; *p* = 0.12). *In vitro* tumor reactivity was determined on day 14 of REP, the potential day of infusion and demonstrated significantly increased cytotoxicity (CD137^+^ fraction 62.9 ± 11.0%; unseparated 47.5 ± 12.6%; *p* ≤ 0.021) (Figure 4C). Although not significant (*p* ≤ 0.23), increased IFNγ secretion was observed in two out of three patients (Figure 4D).

Large-scale expanded TIL were further analyzed for CD4 and CD8 expression, their memory phenotype and expression of co-inhibitory molecules, such as PD-1, TIM-3, and LAG-3 (Table 3). Over 99% of the cells were CD3^+^ T cells. Unseparated TIL and the CD137^+^ fraction demonstrated a similar phenotype (*p* values ≥ 0.4).

**Table 3:** Memory phenotype and expression of co-inhibitory molecules on rapid expanded TIL.

	Patient 1		Patient 2		Patient 3		*p*-Value
	Unsepa.	CD137 fraction	Unsepa.	CD137 fraction	Unsepa.	CD137 fraction	
**Subpopulations**							
CD4	4.7	3.9	51.0	28.3	0.4	0.3	0.69
CD8	95.3	96.1	49.0	71.6	96.6	96.7	0.69
**Memory phenotype**							
T_N_	0.22	0.32	0.88	0.95	0.41	0.27	0.98
T_CM_	1.01	1.14	2.43	3.68	2.85	1.70	0.94
T_EM_	98.7	98.6	89.8	93.4	94.5	96.8	0.56
T_EMRA_	0.04	0.02	6.88	2.02	2.30	1.27	0.40
**Co-inhibitory molecules**							
PD-1	14.3	14.2	32.3	33.0	23.1	20.0	0.88
TIM-3	61.8	63.1	70.9	71.6	76.8	77.4	0.86
LAG-3	4.94	4.79	70.6	63.3	8.35	8.84	0.88

*T_N_ (naïve), CD3^+^CD45RA^+^CCR7^+^; T_CM_ (central memory), CD3^+^CD45RA^−^CCR7^+^; T_EM_ (effector memory), CD3^+^CD45RA^−^CCR7^−^; T_EMRA_ (effector), CD3^+^CD45RA^+^CCR7^−^*.

*Cells were gated on viable, singlet CD3 T cells*.

### CD137^+^ Cells Recognized Shared Tumor Antigens and Neoantigens

**Figure 5:**
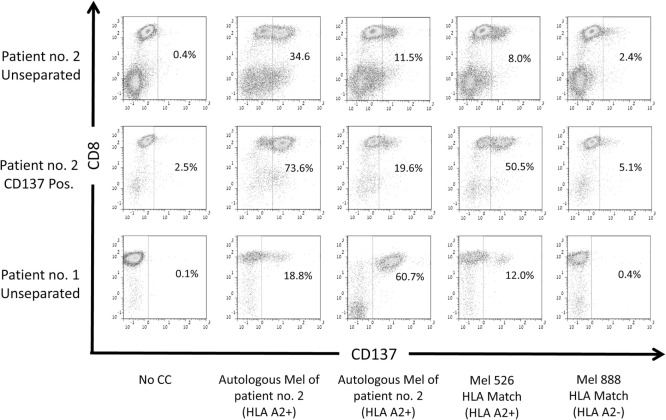
Reactivity of CD137-enriched TIL against shared tumor antigens. CD137 expression of TIL cultures derived from two patients post rapid expansion. Both patients carry the HLA-A0201 allele (HLA-A2^+^). CD137 expression was evaluated before coculture and after a 6-h coculture with autologous melanoma (mel) lines of the patients, HLA-A0201-expressing melanoma line 526 or HLA-A0201^−^ melanoma line 888. Cells were gated on viable, singlet CD3 T cells.

Previously, it has been described that CD137 is upregulated upon coculture with cells that present shared and neoantigenic tumor peptides (31, 32). To evaluate, if the CD137^+^ fraction after REP, ready for infusion, is indeed enriched for TIL directed against shared antigens and/or neoantigens, we analyzed unseparated TIL or the post-REP CD137^+^ fraction of patient #2. Patient #2 was chosen for this analysis, as the patient’s cells express HLA-A0201^+^ and the mutated neoantigenic peptide recognized by the patient’s TIL was known. Unseparated TIL and the post-REP CD137^+^ fraction were cocultured with four different target cells: (1) autologous, low passage melanoma cells; (2) allogeneic, low passage melanoma cells of patient #1 (HLA-A0201^+^); (3) established melanoma line mel 526 (HLA-A0201^+^); and (4) established melanoma line mel 888 (HLA-A0201^−^). As shown in Figure 5, the frequency of CD137-expressing cells was increased post-REP from 34.6% in unseparated cells to 73.6% in the CD137^+^ fraction when cocultured with autologous tumor cells. An increase was also observed after coculture with allogeneic HLA-A0201 matched tumor cell lines (11.5% unseparated to 19.6% in CD137 fraction after coculture with melanoma of patient #1; and from 8.0% to 38.9% after coculture with mel 526). On the other hand, CD137 expression was low after coculture with the HLA-mismatched cells mel 888 (2.4 and 5.1% for unseparated and CD137^+^, respectively). Also when TIL of patient #1 were cocultured with autologous (60.7%) or HLA-A2 matched allogeneic tumor cells (mel patient #2, 18.8%; mel 526, 12.0%) CD137 expression was high, but low after co-incubation with HLA-mismatched tumor cells (mel 888, 0.4%). This indicates that CD137-enriched TIL exhibit the ability of recognizing shared tumor antigens, presented by autologous or allogeneic tumor cells in an HLA-dependent manner.

**Figure 6:**
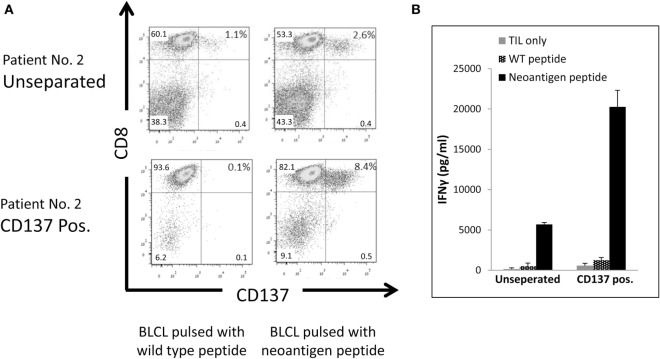
Reactivity of CD137-enriched TIL against neo antigens. Comparison of unseparated and CD137 positively enriched TIL of patient #2 post rapid expansion. TIL were tested after an overnight coculture with autologous B-LCL pulsed with a neoantigen peptide or control peptide at ratio of TIL to B-LCL of 1:4. CD137 expression by FACS. Cells were gated on viable, singlet CD3 T cells **(A)**, and interferon (IFN) γ secretion by enzyme-linked immunosorbent assay (ELISA) **(B)** was tested. ELISA was performed in triplicates.

To evaluate, if the CD137-selected fraction is also enriched for tumor mutation-specific TIL, we determined the neoantigen reactivity of post-REP TIL of patient #2. Comparing WES data of the tumor tissue and PBMC revealed 3,256 non-synonymous cancer mutations, which were narrowed down to 223 potentially expressed peptides following RNAseq. The number of potential neoepitopes was further reduced to 162 after applying the NetMHCpan predication tool. EBV transformed B cell lines of patient #2 were pulsed with 25-mer peptides of each candidate peptide and cocultured with post-REP TIL of patient #2. CD137 expression was determined. Peptide #29 demonstrate increased CD137 expression (2.6%) compared with other peptides (around 1%, not shown) or the wild-type peptide (1.1%) (Figure 6A).

As shown in Figure 6A, following CD137 separation the fraction of TIL recognizing the mutated peptide increase by 3.2-fold from 2.6% in the unseparated TIL to 8.4% in the CD137^+^ fraction. Antitumor reactivity was further examined by IFNγ ELISA. The secretion of IFNγ was significantly increased from 5,687 ± 224 pg/ml in unseparated TIL to 20,264 ± 2,043 pg/ml in CD137-enriched TIL (*p* ≤ 0.01) after cocultured with neoantigen pulsed B-LCL (Figure 6B). These results demonstrate that CD137 bead separation increases the fraction of neoantigen-reactive TIL.

## Discussion

The nature of antigens that allow specific tumor recognition and rejection by TIL cells has long remained obscure. T cells reactive against tumor-specific mutations have been described as the key players for successful tumor regression. In a first clinical trial with neoantigen-enriched TIL, Tran et al. could demonstrate objective response in a metastatic epithelial cancer patient treated with a 95% population of mutation-reactive TIL (16). In addition, neoantigen-specific T cells were identified in the infusion products of melanoma patients achieving a durable response with TIL therapy (18, 19) or in the blood of patients following treatment with immune checkpoint antibodies (20, 21).

Many efforts are being made to identify and isolate tumor-specific T cells that will potentially allow tumor recognition and cancer regression. Detection of rare neoantigen-specific T cells relies on the identification of somatic mutations specific for tumor cells. These mutations exhibit patient-to-patient variability even in the same type of cancer. The process requires advanced technology such as WES, RNA sequencing, *in silico* analysis followed by synthetic peptides or tandem minigenes screening.

The identification of patient-specific, neoantigen-reactive TIL is not only labor intensive, but also requires several weeks in addition to the regular TIL production, which by itself can take up to 2 months. As most of the metastatic melanoma patients enrolled to TIL ACT have highly advanced metastatic disease, this period is unreasonable for many patients. Quicker and easier methods for the isolation of antitumor-specific TIL are therefore required to make this approach clinically applicable (5, 26).

In addition, T cells reactive against shared antigens, such as differentiation antigens in melanoma, have also demonstrated clinical activity. In adoptive cell transfer trials with gp100 or MART-1 TCR-transduced T cells, objective response rates of 19 and 30%, respectively, have been reported (12). These results indicate the importance of finding a biomarker that can identify both, neoantigen and shared antigen reactive T cells.

CD137 is an important regulator of the immune response. The specific antigen-induced expression of CD137 provides an opportunity for the selection of tumor-reactive T cells.

Here, we report a simple, robust and fast method for the enrichment of tumor-reactive T cells for therapy. We describe for the first time a methodology that was designed to address clinical needs and limitations. Every step was validated and optimized. The clinical feasibility of the method was assessed. All reagents used for this process were GMP compliant and approved by the local IRB.

Moreover, the novelty and importance of this study lies in its most detailed and optimized strategy to obtain a T cell infusion product with superior antitumor reactivity, which can be easily embedded in the clinical cell production. CD137 was previously used to identify T cell reactivity against neo-peptides when pulsed on APC, but not as a selection tool for mutation-specific TIL.

Clinical aspects, such as the fact that the number of autologous tumor cells is often limited, the absence of tumor cells in the infusion product or the up scaling of the coculture assay to 200 × 10e6 TIL, were taken into account.

**Figure 7:**
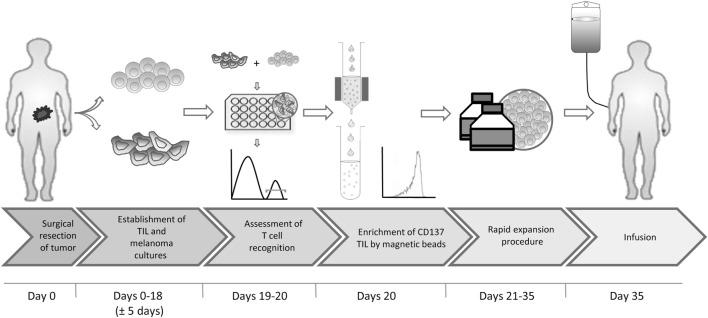
Proposed flowchart.

In short, we propose the following protocol (Figure 7): (1) surgical resection of a tumor lesion; (2) establishment of TIL and autologous tumor cultures (yielding at least 200 × 0e6 TIL). This step requires typically 18 days but may vary between 2 and 4 weeks; (3) transfer of tumor cells to 24-well plates, to allow their adhesion to the plates overnight and addition of TIL at an E:T of 8:1 for 6–8 h; (4) magnetic bead separation with CD137 CliniMACS reagents and MACS ART columns, all CE approved; (5) standard rapid expansion of the CD137^+^ fraction with irradiated feeder cells, anti-CD3 antibody and IL-2, resulting in an approximately 1,000-fold expansion within 14 days; and (6) infusion. Steps 1, 2, 5, and 6 are part of the standard TIL production protocol and require altogether 4–6 weeks. The coculture and magnetic bead separation (steps 3 and 4) just add another 2 days to this procedure, resulting in a total of about 35 days for the entire process.

It is the CD137 selection process itself, which enables the enrichment for tumor-specific T cells. As shown in Table 1, only 30.1 ± 25.9% of the TIL express CD137 following coculture, but nearly all cells express CD137 after the selection. CD137 selection may be especially of importance for patients with a low percentage of CD137-expressing cells. For example, in patient #2 CD137^+^ cells were enriched by over fivefold (from 17 to 88%) by the magnetic bead selection. The data shown here provide strong evidence that CD137-selected TIL demonstrated increased *in vitro* antitumor reactivity and contained a higher fraction of neoantigen and shared tumor antigen reactive T cells. Selection based on CD137 expression enables the identification of tumor-reactive T cells without the need to know the epitope specificity or the antigen type. Further analysis of CD137-expressing T cells may lead to new epitope discovery.

The direct implementation of the CD137 separation method may provide a simple way to improve the objective response rate of TIL therapy. Based on the results shown here, we plan to initiate a clinical trial with CD137-selected TIL in the near future.

## Author Contributions

SS-O, EM-S, and MB designed the study. SS-O, EM-S, OI, and SY acquired the data. SS-O, EM-S, OI, SY, GM, SY, JS, and MB analyzed and interpreted the data. All the authors revised the work, approved the final version, and agreed to be accountable of the work.

## Conflict of Interest Statement

The research was conducted in the absence of any commercial or financial relationships that could be construed as a potential conflict of interest.

## Supplementary Material

Figure S1 Gating strategy.
