# Supplementary figures and images for: Selection of Shared and Neoantigen-Reactive T Cells for Adoptive Cell Therapy Based on CD137 Separation
Source: Front Immunol. 2017 Oct 10;8:1211. doi: 10.3389/fimmu.2017.01211 (PMC5641376; doi:10.3389/fimmu.2017.01211)

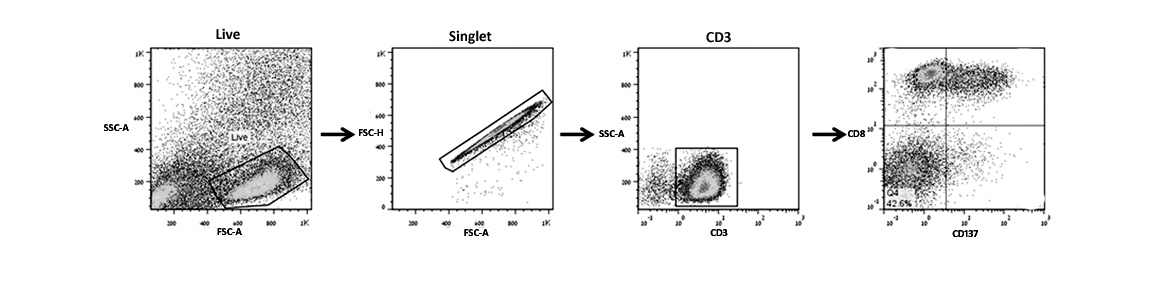

Supplement: Figure S1 — Gating strategy. [file Image_1.TIF]
